# When neurons split the load

**DOI:** 10.7554/eLife.87861

**Published:** 2023-05-04

**Authors:** Itamar Lev, Manuel Zimmer

**Affiliations:** 1 Department of Neuroscience and Developmental Biology, Vienna Biocenter, University of Vienna Vienna Austria; 2 Research Institute of Molecular Pathology, Vienna Biocenter Vienna Austria

**Keywords:** memory coding, neural network, calcium imaging, memory, *C. elegans*

## Abstract

Various aspects of olfactory memory are represented as modulated responses across different classes of neurons in *C. elegans.*

**Related research article** Pritz C, Itskovits E, Bokman E, Ruach R, Gritsenko V, Nelken T, Menasherof M, Azulay A, Zaslaver A. 2023. Principles for coding associative memories in a compact neural network. *eLife* **12**:e74434. doi: 10.7554/eLife.74434.

Memories are composed of the molecular and cellular traces that an event leaves in the nervous system. In turn, these neuronal changes enable the brain to weave together different features of the experience – for example, its outcome – with certain properties of the environment at the time.

Even the small worm *Caenorhabditis elegans,* a tractable and well-studied model organism with 302 neurons, can form such associations. Through conditioning, these animals can ‘learn’ to prefer a stimulus – for instance a smell – that is associated with food being present. This requires neurons to encode information so that an experience (e.g. smelling a specific odor) is correctly linked to valence (whether the situation was positive or negative, depending on the presence or absence of food).

Previous studies have already implicated specific genes and neurons in these processes, for example (Jin et al., 2016; Tomioka et al., 2006). However, this reductionist framework cannot fully capture how different aspects of a memory, such as experience and valence, are represented amongst an entire network of neurons. In *C. elegans*, it is possible to identify many of the neurons in these networks, and to record their activity simultaneously at single-cell resolution. This offers a unique opportunity to directly measure the features of memory traces during perception. Now, in eLife, Alon Zaslaver and colleagues at the Hebrew University of Jerusalem – including Chrisitian Pritz as first author – report the results of an extensive series of experiments which examined how olfactory memory modulates neuronal responses in this model organism (Pritz et al., 2023).

First, the team trained groups of worms to associate a conditioning odor, butanone (diluted in a solvent) with the presence or the absence of food (appetitive vs. aversive conditioning; Colbert and Bargmann, 1995; Kauffman et al., 2010). The protocol was adapted for the animals to form either short- or long-term memories of these associations.

A choice assay experiment then confirmed that in both short- and long-term conditions, appetitive and aversive conditioning respectively increased and decreased the worms’ preference for butanone over another smell (diacetyl). Two control groups were also tested: naive animals that had not been experimented on, and worms that had been through a ‘mock’ training identical to the one received during conditioning, but in the absence of butanone (only the solvent was present).

This experimental design allowed Pritz et al. to systematically isolate and investigate the different factors that influence behavior and neuronal activity. For instance, comparing mock-treated and naive individuals helped to capture the impact of experimental parameters other than smell and valence, such as the worms experiencing starvation.

Next, Pritz et al. used calcium imaging to record the activity of the same set of 24 sensory neurons in conditioned, naive and mock-treated animals exposed to butanone or diacetyl. This revealed that, for these classes of cells, the modulation of neuronal activity in response to the odors was mainly taking place for short- rather than long-term memories. Overall, a large proportion of the sensory neurons studied showed fine changes in activity following conditioning, with a few neuron classes exhibiting a stronger response. Detailed analyses highlighted that each class could encode one or several features of the memories, such as the presence of the odor, valence or a specific aspect of the training process. Mock treatment also impacted the activity of a large proportion of sensory neurons, shedding light on how parameters such as starvation can affect neuronal responses. Overall, these results suggest that the neuronal changes associated with short-term memories are distributed across multiple types of sensory neurons, rather than one class being solely dedicated to capturing a specific element of the response.

To further explore this possibility, Pritz et al. developed machine learning algorithms that could predict the type of conditioning the worms received based on their neuronal responses. The models made better predictions if information from more neuron types (up to five) was provided. Principal components analysis, which helps to pinpoint patterns in large datasets, further supported the idea that different task parameters (conditioning odor, valence, and starvation experience) create distinct activity profiles across the sensory circuit.

Next, Pritz et al. demonstrated that modulation of the sensory neurons also impacted the interneurons that they projected onto, and which relay sensory information to the rest of the nervous system (Figure 1). Three classes of interneurons were examined: while one of them mainly responded to the mock training, the others showed conditioning-specific responses. Unlike sensory neurons, however, all three interneurons could encode both short and long-term memories. While these initial findings are intriguing, additional work on larger datasets is probably needed to confirm whether short- versus long-term memory processes are generally allocated to specific classes of cells.

**Figure 1.**
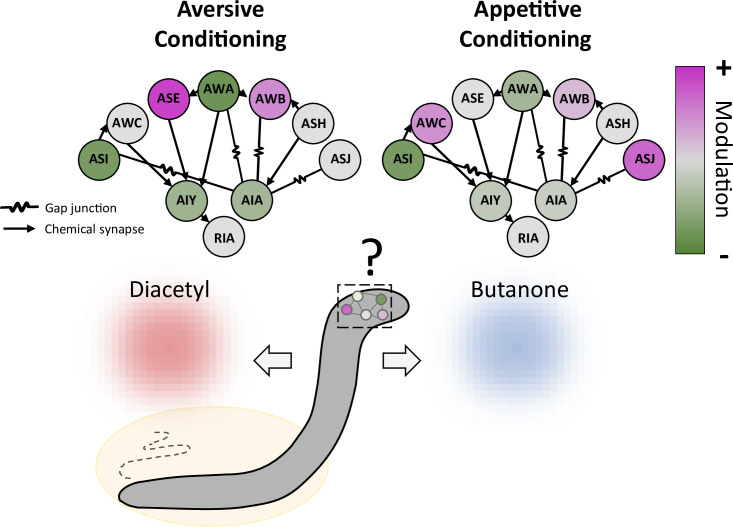
Conditioning induces a distributed modulation of neuronal responses to odor. Worms were exposed to the odor butanone while food was absent (aversive conditioning; left) or present (appetitive conditioning; right); the animals formed memories of these associations, which led them to show respectively decreased or increased preference for butanone over an alternative odor diacetyl. Both types of conditioning modulate a wide range of sensory neurons (circles on outer edge; ASI, AWC and other three letter initials refer to various neuronal classes), which increase (pink) or decrease (green) their activity to varying degrees. The sensory neurons, in turn, alter the activity of three classes of interneurons (AIY, AIA and RIA) that they connect to via gap junctions or chemical synapses. The interneurons then feed information to other parts of the nervous system.

Finally, Pritz et al. used statistical modelling to examine how various sensory neurons shaped the activity of the AIY interneuron, which receives most of its inputs from these cells. The results suggest that AIY modulation was provided by different combinations of sensory neurons depending on the type of training: changes in AIY activity were driven by a single class of neurons after appetitive conditioning, but by a complex circuit of several neuronal classes after aversive conditioning. The memory of different treatment experiences is therefore retained in variable degrees of distribution (the number of classes of neurons involved); whether this could be underpinned by complex changes in the strength of the connections between sensory neurons and interneurons is an exciting hypothesis for future studies.

In conclusion, the work by Pritz et al. adds to existing evidence showing that neuronal signals are distributed across the nervous system in a wide range of organisms – from reactions to stimuli and movement control in *C. elegans*, to memory in animals with larger brains (Lin et al., 2023; Kato et al., 2015; Owald and Waddell, 2015; Tonegawa et al., 2015). As research on *C. elegans* can now also examine larger neuronal networks, it should provide new insights into how the nervous system computes and yields behavior in this and other animals. Advanced machine learning algorithms could help in this effort, as they are uniquely placed to ‘decode’ the signals embedded in large neural activity datasets – for example, which odor a worm is smelling. However, the way that algorithms process that information does not necessarily match the underlying neuronal mechanisms and biological processes accurately. Addressing these problems will require further developing computational and experimental approaches alongside one another.
